# Prematurity, a significant predictor for worse outcome in viral bronchiolitis: a comparative study in infancy

**DOI:** 10.1186/s42506-019-0015-8

**Published:** 2019-03-22

**Authors:** Noussa R. El Basha, Huda Marzouk, May M. Sherif, Amani A. El Kholy

**Affiliations:** 1 Department of Pediatrics, Faculty of Medicine, Cairo University, 2 Atteia Abd El Hadi St., El Maadi, Cairo, 11562 Egypt; 2 Department of Clinical Pathology, Faculty of Medicine, Cairo University, 2 Atteia Abd El Hadi St., El Maadi, Cairo, 11562 Egypt

**Keywords:** Bronchiolitis, Viruses, Preterm, Severity

## Abstract

**Background:**

The rate of admissions to hospital with bronchiolitis has increased over the past years. The reasons for this are likely to be multifactorial including improved survival of preterm infants.

**Aim:**

To assess the severity of viral bronchiolitis in preterm compared to term infants admitted at a tertiary hospital in Cairo, Egypt, based on the outcome.

**Patients and methods:**

This prospective study was conducted throughout a 3-year period from September 2011 to October 2014. It included 153 infants, 74 healthy preterm, and 79 healthy term infants admitted with clinical diagnosis of bronchiolitis at a tertiary hospital in Cairo, Egypt. Bronchiolitis severity score (BSS) was recorded, and nasopharyngeal swabs were obtained from each patient at the time of presentation. Viruses were identified using reverse transcription polymerase chain reaction (RT-PCR). The clinical course and patient’s outcome were recorded.

**Results:**

This study recorded a significantly more severe BSS for preterm compared to term infants. The preterm group had an increased mean length of hospital stay and oxygen therapy and was more likely to need intensive care unit admission and mechanical ventilation (MV) compared to the term group. The mean (± SD) BSS for infections with h-MPV, RSV, and para-influenza 3 was more significantly severe in preterm compared to term infants. Bacterial co-infection was significantly correlated with severity scoring in both groups.

**Conclusion:**

Prematurity significantly affects the severity of bronchiolitis, and this underscores the importance of early categorization as a high-risk group on their first visit. The physician should be aware that their illness runs a more severe course, even if they have no underlying disorders.

## Introduction

Bronchiolitis is the most common lower respiratory tract infection in children less than 1 year of age and is usually of viral etiology [1]. Human respiratory syncytial virus (RSV) is the most commonly causing virus and identified in 60–70% of hospitalized infant with bronchiolitis [2]. Bronchiolitis by itself accounts for the greatest number of hospitalization in infancy during the fall and winter season [3].

Bronchiolitis is a self-limiting condition but may be life threating causing significant severe illness [4–6], and it is the most common cause of hospital admission for infant beyond the neonatal period with rate varying between 1 and 3%. Furthermore, approximately 10% of hospitalized infants will need intensive care admission [7–10].

The rate of admissions to hospital with bronchiolitis has increased over the past 10 years. The reasons for this are not fully understood and are likely to be multifactorial and include improved survival of preterm infants [11].

Epidemiological evidence revealed that young age (less than 6–12 weeks) and premature birth (less than 37 weeks) are associated with high risk of severe bronchiolitis [7, 12, 13].

The aim of the present study was to assess the severity of viral bronchiolitis in healthy preterm compared to healthy term infants in terms of the duration of hospital stay, oxygen saturation and duration of oxygen requirement, and ICU and/or MV requirement.

## Materials and methods

### Study design and subjects

This prospective study was conducted throughout a 3-year period from September 2011 to October 2014. It included 153 infants in the first year of life with clinical diagnosis of bronchiolitis, according to American Academy of Pediatrics [14], and all were admitted to inpatient departments at the Cairo University Specialized Pediatric Hospital. Seventy-four of those infants were healthy preterm (less than 37 weeks) and 79 infants were healthy term. The following specific groups were excluded: neonates, recurrent wheeze, and association of underlying chronic diseases.

### Ethical considerations

This study was approved by the Cairo University Clinical Research Ethics Committee, and informed verbal consents were obtained from parents of the included children. The study design conformed to the Revised Helsinki Declaration of Bioethics.

## Methods

All patients were subjected to thorough history taking, including demographic data, and also, full physical examination was performed with emphasis on signs of respiratory tract infection. The respiratory severity score that has been used in this study is the modified Tal score. This score ranges from 0 to 12, with a higher score indicating more severe disease. Each score is an aggregate of assigned values ranging from 0 to 3 in categories of respiratory rate, retractions, wheezing, and oxygen saturation in room air [15]. Bronchiolitis severity score (BSS) was recorded for each patient at the time of presentation. Subsequently, during inpatient management, the following data were recorded: the clinical course was observed during the hospital stay including the need for intensive care unit (ICU) admission, need for mechanical ventilation (MV), duration of oxygen therapy, and duration of hospital stay.

### Clinical specimens

Complete blood count (CBC) and C-reactive protein (CRP) were determined at the time of study enrollment for each patient. Oropharyngeal (OP) and nasopharyngeal (NP) swabs were obtained for PCR at the time of presentation and put in viral transport media (VTM).

### Molecular identification of respiratory viruses

Total nucleic acid (TNA) was extracted from the NP/OP swabs by the automated KingFisher Flex Magnetic Particle Processor (Thermo Scientific, Waltham, USA) using MagMAX Total Nucleic Acid Isolation Kit (Cat No. AM 1840, Applied Biosystems, Foster City, USA) according to the manufacturer’s instructions. All the viral targets were amplified using specific primers and probes produced by the Center of Disease Control and Prevention (CDC), Atlanta, USA, and following standard protocol for quantitative reverse transcription PCR detection.

Detection of influenza viruses was conducted in the Cairo University Hospital laboratory and confirmed by the Naval Medical Research Unit No.3 (NAMRU-3) laboratory. The samples were screened for the presence of influenza A and B using the CDC kit for influenza following CDC protocol [16]. Samples positive for influenza A were further subtyped according to the CDC protocol to the following types: pandemic influenza 2009 A(H1N1) and seasonal H1 and H3.

Testing for adenovirus; human parainfluenza viruses 1, 2, and 3 (hPIV); respiratory syncytial virus (RSV); and human metapneumovirus (hMPV) was done at NAMRU-3 laboratory by RT-qPCR using CDC specific primers and probes and following a CDC protocol for the detection of non-influenza viruses. Samples were considered positive to the viral target if the amplification curve crossed the threshold line before cycle 40. All clinical samples had to be positive for the human RNAse P gene (RP), with a Ct value ≤ 37, for validation. A positive control for each virus was added to each run to ensure adequate amplification of the target genes.

### Statistical analysis

Precoded data was entered on the Statistical Package of Social Science Software program, version 23 (IBM SPSS Statistics for Windows, Version 23.0. Armonk, NY, IBM Corp.) to be statistically analyzed. Data was presented using mean and standard deviation for quantitative variables and frequency and percentage for qualitative ones. Comparison between groups was performed using independent sample *t* test or Mann-Whitney for quantitative variables and chi-square and Fisher’s exact test for qualitative ones. Spearman correlation coefficient was used to estimate the association between different quantitative variables. *P* values less than 0.05 were considered statistically significant.

## Results

The study included 153 infants with a clinical diagnosis of acute bronchiolitis in otherwise healthy infants. Seventy-nine of them were term and 74 were preterm infants. The detailed demographic characteristics including risk factors for bronchiolitis of both preterm and term infants are summarized in Table 1. Preterm infants were significantly younger than term infants (*P* value 0.001).

**Table 1:** Demographic characteristics of preterm and term infants with bronchiolitis, Cairo University Specialized Pediatric Hospital

Variables	Preterm infants (*n* = 74)	Term infants (*n* = 79)	*P value
Age in months (mean ± SD)	5.2 ± 4.1	7 ± 4.2	0.001
Sex			
Male	36 (48.6%)	41 (51.9%)	0.747
Female	38 (51.4%)	38 (48.1%)	
History of passive smoking	46 (62.2%)	41 (51.9%)	0.253
Non-exclusive breast feeding	32 (43.2%)	27 (34.2%)	0.319

*P value > 0.05 is statistically insignificant

Clinical findings at the time of presentation, BSS, and outcomes of both groups are shown in Table 2. BSS (Mean ± SD) was significantly more severe for preterm compared to term infants at time of presentation (*P* value 0.045). Also, preterm group had an increased mean length of hospital stay, and mean duration of oxygen therapy compared to term group (*P* value 0.002 and 0.001, respectively). Preterm infants were significantly more likely to need ICU admission (*P* value 0.007) and MV (*P* value 0.033) than term infants.

**Table 2:** Clinical findings on presentation and outcomes of the preterm and the term infants with bronchiolitis

Variables	Preterm infants *N* (%)	Term infants *N* (%)	*P value
Respiratory rate (mean ± SD)	54.7 ± 10.2	47.8 ± 10.5	˂ 0.001
Respiratory distress (RD)			
No RD	10 (13.51)	28 (35.4)	0.003
Grade 1	10 (13.51)	12 (15.2)	0.821
Grade 2	16 (21.62)	13 (16.5)	0.536
Grade 3	30 (40.55)	24 (30.4)	0.236
Grade 4	8 (10.81)	2 (2.5)	0.051
Lethargy	28 (37.8)	9 (11.4)	< 0.001
No drink	34 (45.9)	46 (58.2)	0.147
Seizures	4 (5.4)	2 (2.5)	0.431
Vomiting	16 (21.6)	36 (45.6)	0.002
Oxygen saturation (mean ± SD)	85.4 ± 13.1	90.6 ± 9.7	0.016
Duration of oxygen therapy (mean ± SD)	4.4 ± 1.7	2.9 ± 1.4	0.001
PICU admission	18 (24.3)	6 (7.6)	0.007
Duration of PICU admission (mean ± SD)	3.8 ± 1.3	3.2 ± 0.4	0.186
Mechanical ventilation (MV)	12 (16.2)	4 (5.1)	0.003
Duration of MV (mean ± SD)	3.3 ± 1.0	2.5 ± 1.0	0.128
Duration of hospital stay (mean ± SD)	5.4 ± 1.6	4.0 ± 1.6	0.002
Bronchiolitis severity score (BSS)			
Mean ± SD	9.6 ± 3.8	8.3 ± 3.3	0.045
Severe bronchiolitis	36 (48.6%)	26 (32.9%)	0.051

*P values < 0.05 is statistically significant

Viral screening results are summarized in Table 3. Respiratory viruses were detected in 36 (48.6%) preterm patients and in 53 (67%) term patients with acute bronchiolitis. RSV was the most commonly detected virus in both groups, although, more significantly frequent in term infants (57%) than preterm infants (32.4%) (*P* value 0.003). On the other hand, adenovirus was more significantly frequent in preterm than term infants (16.2% and 5.1%; respectively, *P* value 0.033). Concomitantly with the viral respiratory infection, preterm patients had more frequent concomitant bacterial infections than term despite non-significance (*P* value 0.221).

**Table 3:** Viral PCR results among preterm and term infants with bronchiolitis

Viruses	Viral positive in preterm infants *n* = 36 *N* (%)	Viral positive in term infants *n* = 53 *N* (%)	*P value
RSV	24 (32.4)	45 (57)	0.003
Adenovirus	12 (16.2)	4 (5.1)	0.033
h-MPV	4 (5.4)	1 (1.3)	0.198
Influenza A	8 (10.8)	2 (2.5)	0.051
Influenza B	0 (0.0)	0 (0.0)	–
Para-influenza 1	0 (0.0)	0 (0.0)	–
Para-influenza 3	2 (2.7)	6 (7.6)	0.278
Mixed viral infection	12 (16.2)	5 (6.3)	0.071
Co-infection with bacterial	12 (16.2)	7 (8.9)	0.221

*P values < 0.05 is statistically significant

On comparing BSS of different viral agents in preterm and term infants, the mean (± SD) score for h-MPV, RSV, and para-influenza 3 was more significantly severe in preterm compared to term infants (*P* value 0.003, 0.009, and 0.013, respectively), as shown in Table 4.

**Table 4:** Correlation between screened viruses and bronchiolitis severity score (BSS) in preterm and term infants

Outcome	Bronchiolitis severity score (mean ± SD)		
Virus	Preterm	Term	*P value
RSV	9.7 ± 3.9	7.4 ± 3.1	0.009
Adenovirus	9.5 ± 4.0	10.3 ± 2.6	0.717
h-MPV	11.0 ± 1.2	5 ± 0.0	0.003
Influenza A	10.0 ± 3.4	13.5 ± 2.1	0.212
Para-influenza 3	13.0 ± 0.0	6.7 ± 2.4	0.013

*P values < 0.05 is statistically significant

On correlating bronchiolitis severity score to screened viruses and outcome in both groups mixed viral infection did not correlate with severity scoring of bronchiolitis in preterm and term groups (*P* value. 0.723, 0.537, respectively). However, bacterial co-infection was significantly correlated with severity scoring in both groups (*P* value < 0.001). Also, we found significant correlation between BSS and need for admission. Also, there was significant correlation between severity and duration of hospital stay and oxygen therapy as are shown in Table 5.

**Table 5:** Correlating bronchiolitis severity score to screened viruses and outcome in preterm and term infants

Variable	BSS in preterm	*P* value	BSS in term	*P* value
Screened virus (mean ± SD)				
h-MPV	11 ± 1.2	0.721	5 ± 3.3	0.329
RSV	9.7 ± 3.9	0.676	7.4 ± 3.1	0.015
Adenovirus	9.5 ± 4	0.953	10.3 ± 2.6	0.233
Para-influenza 3	13 ± 00	0.214	6.7 ± 2.4	0.189
Influenza A	10 ± 3.4	0.7	13.5 ± 2.1	0.042
Mixed viral infection	9.8 ± 3.6	0.723	9.2 ± 3.8	0.537
Co-infection with bacterial	13.7 ± 1.0	< 0.001	13.7 ± 2.0	< 0.001
Outcome (r)^a^				
Duration of oxygen therapy	0.653	< 0.001	0.721	< 0.001
Duration of hospital stay	0.644	< 0.001	0.803	< 0.001
Duration of PICU admission	0.304	0.220	0.310	0.550

*P* value > 0.05 is statistically insignificant

*BSS* bronchiolitis severity score

^a^(r) is coefficient correlation

## Discussion

We identified a viral etiology in 48.6% of preterm infants and in 67% of term infants hospitalized with acute bronchiolitis; one or more viral pathogens were detected. RSV was the most frequently isolated respiratory virus in both preterm and term infants with bronchiolitis, and adenovirus was the second most frequently detected virus in the current study. The predominance of RSV is in concordance with the assertion that this virus is the single most frequently identified lower respiratory tract pathogen in hospitalized infants worldwide [17–20]. Although, RSV significantly affects more term infant in this study, yet it is likely to be associated with more severe disease in preterm infants, this observation is important in planning protocols for bronchiolitis prophylaxis and management in such high-risk preterm infants. Similarly, h-MPV and para-influenza 3 infections were more severe in preterm than term infants. So, physicians treating preterm infants with RSV, h-MPV, and para-influenza 3 bronchiolitis should be aware that their illness is more protracted and runs a more severe course than term infants, even if otherwise healthy.

The low prevalence of virus detection among preterm compared to term infants with acute bronchiolitis in this study could be attributed to other respiratory viruses which were not included in this study such as coronaviruses, bocaviruses, and rhinoviruses, and may be also explained by the fact that only patients admitted with severe infections were included in our study.

Although there were no significant differences in the length of stay in PICU and the duration of MV among preterm and term, these preterm patients had more frequent need to be managed in PICU, more frequent need for MV, and more prolonged oxygen therapy and hospital stay than term ones. These findings were in consistence with the study by Howidi et al. [21]. These differences may be explained by smaller airway and/or suboptimal immune response in preterm infants. In addition, the significant difference in age between the two groups could partly explain the higher severity among preterm group.

We found also significant correlation between bronchiolitis severity scoring and need of hospital admission, the same as for the duration of hospital stay and duration of oxygen therapy. This was similar to results reported by Ricart et al. [22] who found that severe bronchiolitis had more mean of hospital stay compared to non-severe and more prolonged duration of oxygen and MV. We documented that preterm infants have a more severe form of illness than term infants where we found that prematurity significantly affects the severity score of bronchiolitis. Increased illness severity in infants who had bronchiolitis was discussed in several researches [1, 23]. In this study, we found that preterm infants were significantly younger than those born term, similar to that reported by Fleming et al. [1].

Mixed viral infection and bacterial co-infection in the present study were more frequently observed in preterm compared to term infants with acute bronchiolitis. However, bacterial co-infection was significantly correlated with severity scoring, while mixed viral infection did not correlate with severity scoring of bronchiolitis, and this was in consistence with what was reported by others [22, 24, 25]. This indicates that infection with multiple viruses in infants is a common situation that does not change the clinical course of bronchiolitis. However, some studies have reported an increased risk for severe bronchiolitis in dual viral infection [26–28] or even in some specific viral infections such as RSV and h-MPV co-infection [29–31].

Although respiratory viruses seem to be related to increased risk of severe disease, our data suggest that prematurity have more specific weight in predicting bronchiolitis outcome. So, understanding host susceptibility and immune response of preterm infants to these viruses, as well as targeting the prevention of infection with these viruses in preterm infant, may have even broader implications than only focusing on their role in disease morbidity.

### Limitations of the study

Limiting this study is a possible underestimation of viral etiologies of acute bronchiolitis because testing for rhinoviruses, coronaviruses, and other viruses was not done, and these viruses were previously detected in such patients [32–35]. Also, the enrolled patients were from a single tertiary institution which is a referral center for many local hospitals, so enrolled cases may be more ill than those at other hospitals. The enrolled patients were inpatients only, while patients with mild respiratory symptoms did not undergo diagnostic testing for viral infections; they were treated in outpatient clinics and this could further direct results towards a more severe population and could influence the scores.

## Conclusion and recommendations

Prematurity is one of the risk factors that significantly affect the bronchiolitis severity, and this underscores the importance of early categorization of these infants as a high-risk group on their first visit. Physicians treating preterm infants with bronchiolitis should be aware that their illness runs a more severe course than term infant, even if they have no underlying disorders.
